# Designing a Clinical Decision Support Tool That Leverages Machine Learning for Suicide Risk Prediction: Development Study in Partnership With Native American Care Providers

**DOI:** 10.2196/24377

**Published:** 2021-09-02

**Authors:** Emily E Haroz, Fiona Grubin, Novalene Goklish, Shardai Pioche, Mary Cwik, Allison Barlow, Emma Waugh, Jason Usher, Matthew C Lenert, Colin G Walsh

**Affiliations:** 1 Center for American Indian Health Department of International Health Johns Hopkins Bloomberg School of Public Health Baltimore, MD United States; 2 Department of Biomedical Informatics Vanderbilt University Medical Center Nashville, TN United States; 3 Department of Medicine Vanderbilt University Medical Center Nashville, TN United States; 4 Department of Psychiatry Vanderbilt University Medical Center Nashville, TN United States

**Keywords:** suicide prevention, machine learning, Native American health, implementation

## Abstract

**Background:**

Machine learning algorithms for suicide risk prediction have been developed with notable improvements in accuracy. Implementing these algorithms to enhance clinical care and reduce suicide has not been well studied.

**Objective:**

This study aims to design a clinical decision support tool and appropriate care pathways for community-based suicide surveillance and case management systems operating on Native American reservations.

**Methods:**

Participants included Native American case managers and supervisors (N=9) who worked on suicide surveillance and case management programs on 2 Native American reservations. We used in-depth interviews to understand how case managers think about and respond to suicide risk. The results from interviews informed a draft clinical decision support tool, which was then reviewed with supervisors and combined with appropriate care pathways.

**Results:**

Case managers reported acceptance of risk flags based on a predictive algorithm in their surveillance system tools, particularly if the information was available in a timely manner and used in conjunction with their clinical judgment. Implementation of risk flags needed to be programmed on a dichotomous basis, so the algorithm could produce output indicating high versus low risk. To dichotomize the continuous predicted probabilities, we developed a cutoff point that favored specificity, with the understanding that case managers’ clinical judgment would help increase sensitivity.

**Conclusions:**

Suicide risk prediction algorithms show promise, but implementation to guide clinical care remains relatively elusive. Our study demonstrates the utility of working with partners to develop and guide the operationalization of risk prediction algorithms to enhance clinical care in a community setting.

## Introduction

### Background

Some of the biggest successes in suicide prevention have come from populations with the greatest needs, including Native American communities. The White Mountain Apache Tribe in Arizona has been a leader in this field with their award-winning program surveillance and case management program, called Celebrating Life (CL). After a spike in youth suicides in 2001, tribal leaders leveraged sovereignty and mandated a community-wide suicide surveillance system [1]. Since then, all people working or living on the reservation are required by law to report incidents of suicidal ideation, attempts, deaths, nonsuicidal self-injury, and high-risk substance use, as defined by high-risk patterns of alcohol and drug use, particularly for youth and adolescents in a central registry. Each of these reports is then followed up on in person by trained Apache case managers.

The registry, brief contact, and case management system comprise the backbone of the CL program. International evidence supports this model as a promising approach to reduce the number of people who die by suicide [2]. CL also incorporates more upstream suicide prevention efforts, such as brief culturally informed interventions delivered to children and families at their homes or in schools [3,4], gatekeeper training programs, and door-to-door campaigns. CL has contributed to reducing suicide rates by 38% and suicide attempts by 57% on the Fort Apache Indian Reservation [5]. Given its success, several tribes are in the process of adapting and replicating CL to their own settings.

As awareness of the surveillance and case management programs has grown, so has the volume of referrals. Reaching all those reported to be at risk of suicide and associated behavior is challenging in settings with high demand and large geographies covering hundreds of square miles. Therefore, prioritization is necessary. Currently, the prioritization of cases for in-person follow-ups is based on the severity of the incident behavior reported and the age of the individual. Case managers first try to see clients with a reported suicide attempt, followed by nonsuicidal self-injury, then ideation, and finally, high-risk substance use. If the client has more than 1 referral for an attempt, then case managers use the date of the reported event as another layer of prioritization [1]. This prioritization model attends to those with the most severe reported behaviors, but it does not consider the long-term risk of being suicidal.

Case managers generally rely on in-person interviews or questionnaires to assess the suicide risk of individuals already identified as at risk in the community and who are reported to the surveillance system. Administering assessments requires time, training, and mastery of the case manager role. The reliance on face-to-face approaches to identify someone at heightened risk of suicide is generally the standard practice, yet recent evidence suggests that such assessments may not be insufficient to identify who is at risk and when [1,6]. What drives someone to attempt to die by suicide is complex, yet current methods for risk detection are relatively simple, combining limited factors (eg, 5 questions) in simplistic ways (eg, sum scores) [7]. Despite decades of research, psychiatrists’ ability to identify those at risk is only slightly better than chance [8]. There is growing recognition that methods and models that account for greater complexity are needed to advance suicide prevention efforts [9].

Machine learning applied to suicide risk identification is a promising approach to address this complexity. Machine learning is the application of algorithms to data to gain insight into meaningful patterns that are often difficult for humans to recognize [7]. Recent work applying these methods to suicide prevention shows both promising and potential challenges. The results from several individual studies have reported an increase in predictive accuracy using artificial intelligence [9,10]. However, a recent meta-analysis indicated that machine learning models also have limitations, including low positive predictive values [11]. This is likely a result of the low prevalence of suicide in the general population. However, others have argued that despite low positive predictive value, machine learning algorithms still hold significant promise because of their low cost and overall net benefit [12]. These methods are also thought to be more easily scaled because they rely on electronic data and computing power, both of which are increasingly available. Instead of relying on specialists to conduct assessments, data can be passed through an algorithm and digitally convey a level of risk for future suicidal behaviors.

### This Study

Despite the promise of improved accuracy and potential for scalability, implementing risk algorithm implementation as a clinical tool remains rare. Risk algorithms may be useful, but they are certainly not sufficient to prevent suicide alone. It is critical for any algorithm to be optimized in the setting in which it will be used [7]. In 2017, the White Mountain Apache Tribe and Johns Hopkins Center for American Indian Health (JHCAIH) collaborated to develop and validate a machine learning algorithm to help identify people reported to CL who were most at risk for suicide death or attempts [13]. In this study, we aim to understand how to implement this algorithm to inform care. To answer this question, we used qualitative input from case managers and supervisors to explore (1) how they consider and evaluate risk, (2) how they prioritize cases, (3) what could be done for different levels of risk in their communities, and (4) how the algorithm should be implemented in their workflow? The results of this project informed the implementation of the said risk algorithm into practice, helping case managers to identify and attend to those most at risk of dying by or attempting suicide.

## Methods

### Overview

This project is nested in two larger projects, one of which is the Southwest Hub for Youth Suicide Prevention, focused on youth suicide prevention in Native American communities (National Institute of Mental Health [NIMH] U19MH113136-02S3). The Southwest Hub includes a research study and a public health practice approach that supports 5 other tribes in the southwest and in Montana to implement CL in their settings. The second study, Sustainability of Suicide Prevention Programs in Native communities, focuses on understanding the implementation and sustainability of these surveillance and case management programs, as they are scaled to other tribal partners (NIMH K01MH116335). The focus of this manuscript is to implement the machine learning algorithm within these suicide surveillance and case management programs to help case managers identify and respond to risk. For this study, qualitative in-depth interviews (IDIs) were conducted with case managers from 3 communities implementing CL. The institutional review board at Johns Hopkins School of Public Health and Navajo Nation both determined this project as exempt from oversight because it did not qualify as human subjects research. The White Mountain Apache Tribal Health Board approved this project at the time of grant submission.

### Existing CL Workflow

The existing structure of the CL workflow has been described in detail elsewhere [1]. Briefly, when a referral occurs, the CL staff fill out an intake form (called the *yellow form*). This form includes demographics and basic information on the reportable behavior. Following the intake form, CL case managers attempt to locate each individual. Prioritization of who to find first has been described earlier. If contact is made, during the follow-up visit, case managers gather more information on a follow-up form (called the *pink form*), confirm the behavior, and provide referrals and additional resources. The follow-up form assesses the circumstances around the event and the relevant risk and protective factors. This information is stored in a secure web-based portal.

### Study Participants

Given the aim of the study to obtain insight and input from case managers, a purposeful sampling strategy [14] was used to recruit participants. Participants were eligible to be interviewed if they were current case managers from 3 communities (the White Mountain Apache Tribe and 2 sites in Navajo Nation that serve rural populations) where the CL system was implemented. All staff members were notified of the opportunity to participate and were free to decline. A total of 9 case managers and supervisors participated in 8 IDIs (one IDI was conducted with 2 staff members simultaneously as a joint interview). All participants were employees of the JHCAIH and represented all possible case managers and supervisors in each community.

### Data Collection and Management

IDIs were conducted by a female JHCAIH research associate with a master’s degree in public health and with experience in qualitative data collection and analysis. The interviewer works across a number of suicide prevention projects and is familiar with participants through collaboration with CL and other projects. The interviewer was asked to conduct these interviews by the lead author, so they did not have any particular interest in this topic. Participants were approached for the study through face-to-face meetings. IDIs took place in quiet, private office settings and lasted approximately 30 minutes on average. None of the participants refused to participate or dropped out. We developed an interview guide for IDIs to elicit information that could inform the primary research aim of understanding CL staff perceptions and evaluation and response to risk as well as ideas for how to incorporate a risk algorithm into their work and caseload management (Multimedia Appendix 1). IDI questions covered CL staff’s daily work experience, how they evaluate various levels of risk and what resources and responses are used for individuals at risk, what factors inform their assessments of suicidality, and ideas for when and how a risk algorithm could be most useful to them. Although the development of our guide was not directly informed by an implementation science framework, our approach overlaps with an exploration of the intervention characteristics, inner setting, and characteristics of the individual domains of the Consolidated Framework for Implementation Research (CFIR) [15]. Other domains in the CFIR were not explored directly in the interview guide questions. IDIs were audio recorded, transcribed, and deidentified. Once transcripts had been checked for accuracy by the interviewer, audio recordings were deleted. All files were stored on a secure electronic server, and access was password protected.

### Qualitative Data Analysis

Consistent with methodological approaches to establish the trustworthiness of thematic analysis, data analysis of the transcripts was an iterative process [16]. A preliminary codebook of a priori codes was developed based on the interview guide. A priori codes included codes designed to capture concepts, such as surveillance system experiences, definitions of suicide risk, and risk flag utility. Furthermore, 2 researchers reviewed all transcripts and independently performed in-depth vertical analysis [17] of 2 transcripts to elicit emergent codes from the transcripts. The 2 coders reviewed each code from the 2 transcripts and discussed their disagreements. This review process led to enhanced definitions of each a priori code, a set of emergent codes, and improved consistency between coders. Emergent codes captured important relevant concepts such as program implementation challenges, resource use, and local causes of suicidal behaviors. Iterative discussion among the coders and the lead author supported the revision and development of a final codebook that included a set of 27 a priori and emergent codes. Additional emergent codes were added during the final coding process by each coder and discussed as a team. Dedoose (version 8.3.10, SocioCultural Research Consultants, LLC) [18] was used to apply the finalized codebook to all 8 transcripts (each coder coded 4 transcripts). A coding report was developed by the 2 coders by compiling all pieces of coded text under their respective codes. The analysis team (ie, 2 coders) then examined the consistency of the coded text and discussed any discrepancies that arose. Discrepancies were resolved through consensus agreement. The final coding report included general summaries for each code and the selection of the most representative quotes. As a final step, the coding team organized codes, their summaries, and their representative quotes into broad thematic categories. Qualitative results were then synthesized to inform the implementation of algorithms and associated care pathways.

## Results

### Participants

A total of 9 case managers completed the IDIs. All (9/9, 100%) participants were women. The case managers from White Mountain Apache Tribe all had over 2 years of experience, whereas the case managers from Navajo Nation had less than a year of case management experience. A total of 33% (3/9) of participants had master’s degrees, whereas the other case managers (6/9, 67%) had bachelor’s degrees.

### Qualitative Data Results

The results are organized into four thematic categories: (1) planning and prioritization of follow-up visits; (2) suicide risk, definition, and causes; (3) interventions and responses; and (4) considerations for risk flags and algorithms. We report detailed findings under these 4 thematic categories and how this information was used to inform the algorithm implementation and care pathways.

### Planning for Follow-ups and Prioritization of Cases

**Figure 1:**
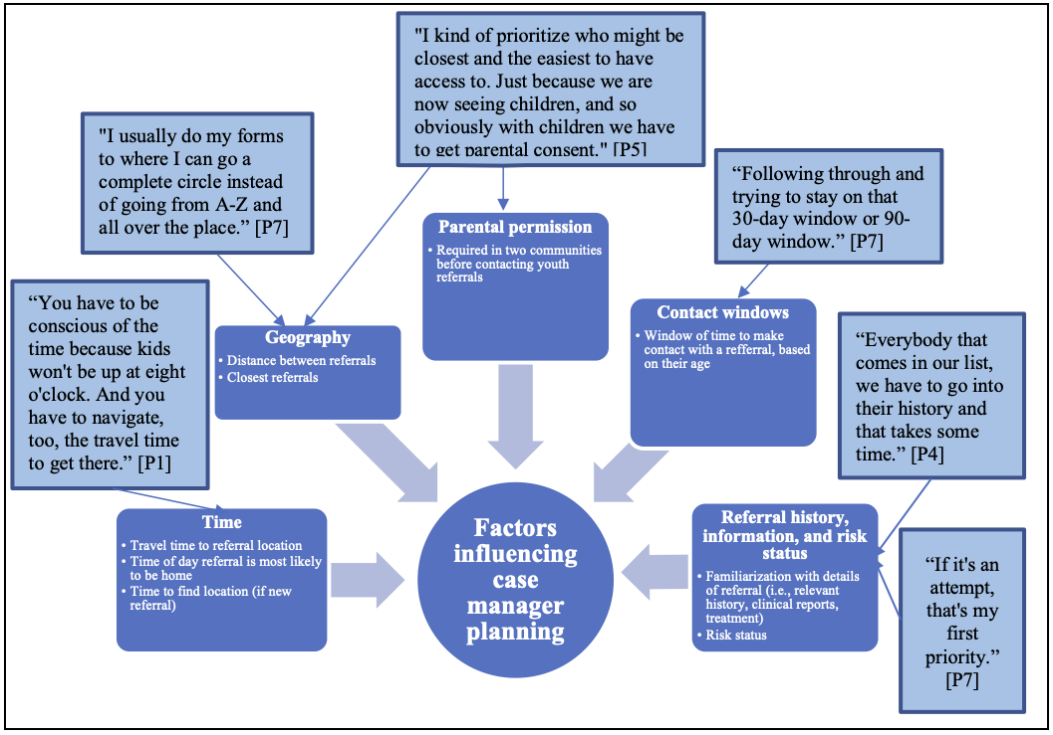
Factors influencing case manager planning and representative quotes.

Participants described how they plan their workdays, keep track of referrals and follow-ups, and schedule subsequent visits. The factors that case managers consider when planning their days are illustrated in Figure 1. After risk status, geography and time were important considerations. For example, case managers considered how long it takes to reach a person’s location, including how much time is needed to physically find an individual. Home addresses on reservations are often unreliable and, in some cases, do not exist. Finally, case managers also considered the date the referral was made, as there are reportable time windows in which a follow-up visit should be completed.

Case managers primarily use current behavior and a person’s known history to make decisions about the order of follow-up visits. According to existing protocols, reported suicide attempts are the top priority, followed by intentional self-harm, suicidal ideations, and high-risk substance use. Some participants noted that high-risk substance use on its own could often be youth experimentation, but that high-risk use in conjunction with suicidal ideation would raise their level of concern. A history of previous suicidal risk behavior is also a factor for consideration, although more experientially, where case managers might be familiar with an individual, rather than having documentation of an individual’s behaviors over time. One case manager (P7) noted the amount of time it takes to look into a person’s history, “Everybody that comes in our list, we have to go into their history and that takes some time.”

Prioritization of cases based on risk status also interacted with factors such as time and geography. High-priority cases are seen first, but other less risky cases that are nearby may be checked on: “If you’re going into one area, you’re going to do the priority, there’s more people that are within that little radius, you’re going to try and hit those then go to the next priority area” [P1].

Other staff (P5) indicated that sometimes geography and time are more of a priority than risk status: “I kind of prioritize who closest and the easiest to have access to.” Staff in all communities discussed encountering unexpected challenges that disrupt their plans each day, such as being unable to locate a residence or attempting a follow-up, but finding their intended client is not home. To overcome some of these challenges, the participants outlined how they collaborated with community partners. For example, in one community, community-based chapter houses that are similar to local town halls represent a valuable local resource that supports case managers in locating and learning about referrals: “If there’s no house description, of course, there’s a physical description or location or address on the referral system, so a lot of times I go to the Chapter houses because they’re a great resource for me” [P3].

### Suicide Risk: Causes and Definitions

Participants outlined some of the factors that contribute to suicide in their communities, including sexual abuse, substance use problems, stress, and lack of family support. Some described how limited access to education compounds family problems and difficult home environments to make life more difficult, which can lead to substance use as a way to cope with feelings of despair and suicidal thoughts. Participants characterized the connection between substance use and suicidal behavior as an indication of someone who might be at long-term risk of suicide:

It comes back to drugs and alcohol. Kids feel neglected; that’s why they feel suicidal. Under the influence of pressure of drugs and alcohol, they get involved, they get hooked.

P8

When asked about how they assess a person’s risk status, participants generally agreed that each case and situation varied and must be evaluated in context. Case managers described the ability to observe a person’s level of risk when talking to them, including their attitude, body language, and reactions. Although we asked about signs and indicators of high, low, or medium risk, case managers only described the risk in terms of high or low risk, and not on a continuum. Textbox 1 outlines the factors that participants described as signaling higher or lower risk for suicidal behaviors. Factors included participant behaviors in the moment (ie, crying), reported risk factors such as feeling currently unsafe or lacking a support system, and past history of risky behavior. For example, one participant (P5) said, “Especially if you start to notice, maybe their environment is not safe or it’s unhealthy, then that definitely puts them at more risk.”

**Factors indicating higher or lower risk of engaging in suicidal behaviors.**

**Factors Indicating Higher Risk**

- Multiple risky behaviors (eg, high-risk substance use and suicidal ideation)
- Share openly and agree to wellness checks
- Crying or tearful
- Have problems with substance use
- Lack a family support system
- Report recent suicidal ideation
- Have been referred multiple times
- Have a history of suicide attempt or attempts
- Report feeling unsafe
- Live in an unhealthy home environment

**Factors Indicating Lower Risk**

- Acknowledging that an act occurred and attributing it to a spur of the moment mistake
- Indicating a lack of current suicidal ideation when asked

Some disagreement arose in relation to referrals who were very open about their experiences compared with those who denied the occurrence of the event. For example, one case manager stated as follows:

The ones that are more high-risk are the ones where I notice...they’re the ones that are pretty open about it. They’re the ones that want to talk and they’re the ones that will tell me what’s going on...The ones that I know are high-risk usually agree to those wellness checks.

P3

Other participants felt that denial of an act was an indication of increased risk. Receiving repeat, multiple referrals was also viewed as an indicator of higher risk, but participants also noted that some suicide deaths have occurred in people who were never referred to the system and had no obvious indicators of being at high risk.

### Interventions and Responses

**Figure 2:**
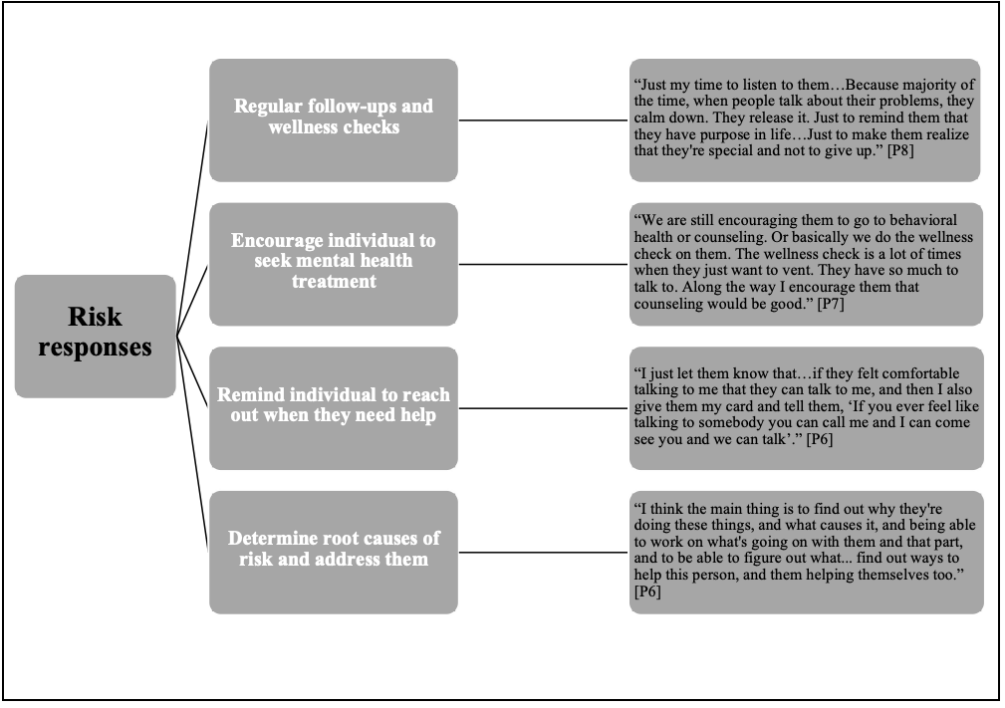
Responses to long-term suicide risk.

Participants described responses for those at long-term risk (ie, they are at risk, but behavior is not imminent) and those at more urgent risk (ie, suicidal behavior is imminent). The main response for those at more long-term risk was facilitating connections for individuals to local resources and services to support their mental health and well-being. Within existing CL resources, case managers can offer brief contact interventions in the form of regular wellness checks, short psychosocial interventions related to suicide prevention and substance use, support access to counseling when they encounter someone who is at risk, and provide support services to families who have lost a member because of suicide. The responses specific to suicide risk are shown in Figure 2.

When responding specifically to an urgent risk, participants described offering immediate support such as transport to counseling services or the emergency department:

I’ll tell them right then and there, ‘do you feel like you want to speak to a counselor, because I’m here and I can give you a ride, I can sit there and help you fill out forms, I can sit there, and I can support you.’

P7

If necessary, case managers described protocols to call the police for support by transporting people at urgent risk to the local emergency department. For less severe urgent risk, the response involves safety planning, attending to acute risk factors through referrals to local services, making sure individuals are aware of what resources they can reach out to, and scheduling wellness checks.

### Risk Flags and Algorithms

Many participants agreed that generated risk flags indicating individuals with high priority or risk would be useful in planning and case prioritization:

That would be great...would be very helpful, especially in our prioritizing so that, we know that we’re accurate, we’re not missing people or anything.

P2

Participants also felt it would be ideal to learn about a risk flag as soon as possible to initiate immediate follow-up, particularly if a person is flagged for being at long-term risk. When following up with individuals who have been flagged by the algorithm, some participants suggested that additional, separate follow-up should be carried out with flagged individuals based on current follow-up protocols and geared toward obtaining more information to facilitate better care and monitoring. The existing protocols allowed for wellness checks as desired by the individual, but some case managers felt that these should be mandated as a way to provide more ongoing support. This was considered a way to use resources effectively and efficiently if provided to those at the highest risk and would provide a more uniform approach to follow-up care.

Some participants were less sure about the potential utility of risk flags but suggested risk flag reports should include as much information as possible to help build case managers’ trust in the algorithm because they could compare it with the factors and flag using their own judgment to assess the algorithm’s accuracy. Knowing more about why the algorithm generated a flag would also support case managers in explaining the surveillance system and risk flags to community partners, building trust across collaborations. Participants suggested that risk flag reports should try to convey an individual’s history and the statistical chance that they might exhibit dangerous behaviors in addition to the reasons they were flagged:

I think it would be great to have an algorithm that does flag high risk individuals, if alcohol, drug use, higher risk factors, domestic violence, sexual violence...what are the risk factors of getting flagged, and that would be great to see and see if there is a correlation between the actual data that we’re putting in and knowing those individuals whether or not are higher risk, and seeing how it actually pops up and the algorithm to see how it correlates.

P2

The need for information to accompany risk flag reports was underscored by participants noting that integrating knowledge of an individual’s history is essential: “How can we help someone if we don’t know their history?” [P9].

### Qualitative Results Synthesis to Inform Implementation

Our findings related to the four broad thematic categories of (1) planning and prioritization of follow-up visits; (2) suicide risk, definition, and causes; (3) interventions and responses; and (4) considerations for risk flags or algorithms that help inform the implementation of algorithms and associated care pathways. First, findings from planning and prioritization of follow-up visits demonstrated the importance of understanding a person’s history when prioritizing for follow-up care. Leveraging historical records on the individual to identify future risk status using the algorithm expedites this process. The algorithm itself was designed with implementation in mind using simple mathematical formulas based on responses to items on a data collection form that asks about the individual’s history and current circumstances [14]. Our findings from thematic category 2, suicide risk, definition, and causes, clearly showed that all case managers thought of risk as dichotomous. This informed how we operationalize the continuous probability score to produce a dichotomous risk status. Case managers also brought up several considerations they use in determining the risks that are captured through clinical observation such as crying or observations of the living situation. On the basis of this information, implementation of the algorithm had to ensure a way for case manager clinical judgment to factor into the classification of risk status.

For the interventions and responses theme, our findings suggest that there were numerous approaches that the case managers could implement depending on risk status without the introduction of new intervention approaches. Given that brief contact intervention in the form of wellness checks is already part of the program, albeit an optional addition if the individual expresses interest, and evidence supporting the effectiveness of regular contact with individuals to reduce risk [19,20], program leaders decided to mandate regular wellness checks to ensure that those deemed to be at high risk would receive continued contact with staff. This brief contact approach could also be combined with other evidence-based interventions, such as safety planning and brief psychosocial interventions, that staff already have experience in providing.

Finally, the findings from the theme of broad considerations for the implementation of risk flags and algorithms corroborated the importance of considering an individual’s behavioral history in the approach, while also emphasizing the importance of timeliness of the notification of risk status and the importance of trustworthiness of the risk status. On the basis of these findings and the need to get this information to the case manager as soon as possible, the algorithm is programmed into the follow-up data collection form. The case manager can use this on a mobile device, and a notification automatically informs them of risk status at the end of the visit. A report of all high-risk cases is also generated and reviewed on a biweekly basis to ensure timely follow-up. Regarding trust in the algorithm, a dichotomous score was selected to maximize the diagnostic specificity. This was done to ensure that the risk flag was not flagging individuals who were clearly not at risk. The favoring of diagnostic specificity with the algorithm was only done in the context of our theme 2 findings that showed how case managers could enhance diagnostic sensitivity through clinical evaluation of the person and circumstances.

**Figure 3:**
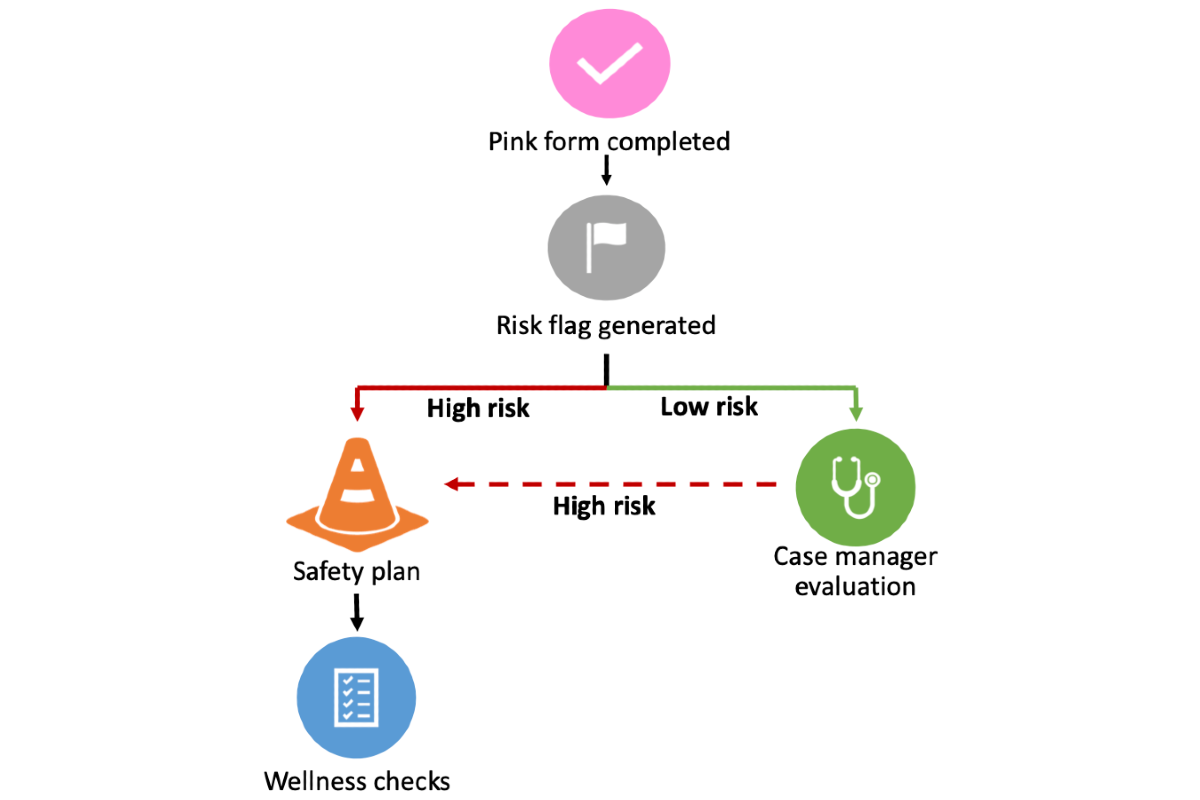
Clinical decision and appropriate care pathways tool. Pink form is the name for the follow-up form that is used at the follow-up visit.

Together, the information from our findings is depicted in the process flow chart in Figure 3. First, the CL system receives an intake form, and a case manager attempts to locate the individual to follow up with them. When the case manager makes contact, the follow-up form (or *pink form*) is completed. This pink form incorporates information about the individual’s past as well as the current circumstances and circumstances around the reported event. The risk flag is generated at the end of the pink form, immediately notifying the case manager of the person’s risk status. Biweekly meetings are held to review these cases, as well as any other cases determined to be at high risk by the case manager. Finally, all high-risk cases receive mandated longitudinal wellness checks in concordance with evidence-based brief contact interventions.

## Discussion

### Principal Findings

This study sought to understand if and how a suicide risk prediction algorithm could be used to inform care provided by paraprofessional case managers to those at risk of suicide. We designed this study to inform the implementation of the risk prediction algorithm in CL suicide surveillance and case management programs. Case managers indicated that they consider several factors, including current behavior, past history, and geographic location, to help them prioritize the individual to be followed up first. Suicide risk was thought of in dichotomous terms with many interrelated factors indicating higher risk and fewer factors indicating lower risk. Acute or urgent risk was addressed through immediate support and transportation or consultation with emergency services. For individuals who were at a higher risk, but not in need of emergency services, case managers highlighted the importance of several responses that could be provided within the constraints of existing resources, including regular wellness checks, encouraging and supporting the individual to seek mental health treatment, and reminding the individual to reach out for help.

Most case managers agreed that an additional tool to help them identify and prioritize high-risk cases would be useful. They expressed an interest in the algorithm producing a dichotomous result that was timely and highly trustworthy. This indication would then guide them in providing an appropriate care pathway that was compatible with existing resources. Taken together, the results of this study informed the clinical decision support (CDS) and the corresponding care pathways. Each individual is flagged as high-risk or low-risk after the completion of an in-person follow-up. If the person is flagged as high-risk, the case manager provides regular wellness checks for that individual. If the person is flagged as low -isk, no additional procedures are performed, unless the case manager determines otherwise. These procedures are now being implemented in partnership with the White Mountain Apache Tribe.

Despite robust interest, machine learning models have rarely been translated into clinical care [21]. In recent years, there has been a proliferation of suicide risk prediction models [11,13,22-24], but the implementation of these models has been much more limited. The Veterans Health Administration’s Recovery Engagement and Coordination for Health-Veterans Enhanced Treatment program has had some early success implementing predictive models and associated care into their suicide prevention efforts [25]. Veterans Health Administration’s Recovery Engagement and Coordination for Health-Veterans Enhanced Treatment is focused on the initial identification of high-risk individuals from a population-based sample. In contrast, our model is aimed at prioritizing outreach and follow-up care to those already identified as at risk. This was an important distinction. More work is needed to further explore whether our model and associated care pathways are appropriate for initial risk identification and care. Although our model incorporates some past historical features, it draws primarily on structured information collected by the case manager. This is in contrast to many electronic health record–based models that have been developed that draw on existing variables in records that are not readily available or asked about by clinicians. This difference in approaches was primarily driven by computational barriers and the feasibility of implementing the model given the existing information technology infrastructure. Notwithstanding, to the best of our knowledge, this study was among the first to adopt a qualitative approach to guide the implementation of a suicide risk prediction algorithm in a clinical- and community-based care setting. The use of qualitative methods, including user-centered design methods, has been used for other decision support tools, including those related to gun safety and suicide [26] and in-hospital clinical deterioration [27].

Several key challenges in suicide prevention emerged from these qualitative interviews. First, providing these services in rural and high-poverty areas is challenging. Case manager participants reported difficulty in finding clients, not having addresses, and driving long distances to ensure in-person follow-up. Tools that could help with streamlining driving routes and prioritizing cases within those routes may be helpful. There was also confusion about what indicates risk—clients are either open about their experiences or deny them, and these two reactions indicated different levels of risk to different case managers. Clinical judgment is valuable in determining the risk of suicide but is insufficient [28]. Explicitly valuing case managers’ clinical judgment was critical, but given conflicting interpretations and differing levels of experience, the addition of an algorithm to aid these decisions was seen as valuable.

Case managers also raised some issues that we were unable to address in our CDS design. For example, case managers expressed a desire for complete transparency in what the algorithm used to calculate a risk score and how that score is computed. For example, a case manager stated, “...see if there is a correlation between the actual data that we’re putting in and knowing those individuals whether or not are higher risk and seeing how it actually pops up and the algorithm to see how it correlate.”

Although we were able to consider the broad importance of trustworthiness, we were not able to fully comply with the specifics of this need, given constraints on the underlying data collection platform and the amount of time it would take to process this information for each individual. Future work will continue to explore this issue with case managers as the CDS is implemented. Although stakeholder opinions are critical to designing tools that work in practice, other considerations, including the underlying computational infrastructure and organization and care context, are critical to consider in the design and implementation process of any such tool.

Although we did not use a specific implementation science framework to guide our study, the themes that arose in our study are consistent with several constructs in the CFIR [15]. For example, themes that emerged around the intervention characteristics included the relative advantage of the algorithm with the existing standard of care and the considerations of the complexity of the approach and the need for the algorithm to produce a dichotomous indicator to enhance interpretability. The domain characteristics of the individuals also emerged in our data, particularly around the knowledge and beliefs about the intervention being critical to successful implementation. Finally, themes related to the outer setting emerged as well. Cosmopolitanism, or the need to network with other organizations to help find individuals, was considered critical. The importance of external policies and incentives also increased. This was particularly related to the need for parental permission before contacting youth in two out of the three settings, which was discussed as a challenge confronted while implementing the program. Our methodological approach was focused more on the intervention and direct implementers of CL, given the narrow focus on how to operationalize the algorithms. However, other themes, particularly those related to the outer context, emerged as important factors to consider when broadly implementing CL-type programs and predictive analytics in practice.

### Limitations

We interviewed all case managers employed at the time of the interviews for this study, as qualitative feedback from them would be highly relevant to inform local implementation. However, the sample size was small and limited the transferability of our findings to other contexts. We also may not have reached saturation through sampling. Further work could continue to explore these themes with other case managers as they become available to understand if more data collection and analyses are warranted. One interview was conducted with 2 participants simultaneously, which could have limited their ability to provide feedback in the same way as the other participants. We were unable to explore differences in interviews based on the experience of the case managers, as our sample size was small and many of the potential differences could be confounded with relative differences in the length of time that the programs had been implemented at each site. Finally, our participants did not have experience using the algorithm, which meant that their responses were based on a hypothetical situation. Their views on the algorithm, its utility, and its implementation may change over time—views that would be important to capture to ensure ongoing successful implementation.

### Conclusions

Careful thought and planning should be put into implementation efforts to fully realize the potential of suicide risk prediction algorithms. To our knowledge, this study is among the first to use qualitative methods to study implementation considerations for a suicide risk prediction algorithm in a community context. Our findings guided the development of CDS and associated care pathways. These findings inform the implementation of the algorithm to enhance clinical care for individuals at risk of suicide. This body of work also reflects tribal communities’ commitments to innovative, efficient, and effective solutions to reduce suicide in native communities with the potential to scale to other communities in need.

## Appendix

Multimedia Appendix 1 In-depth interview guide.

## Abbreviations

CDS: clinical decision support
CFIR: Consolidated Framework for Implementation Research
CL: Celebrating Life
IDI: in-depth interview
JHCAIH: Johns Hopkins Center for American Indian Health
NIMH: National Institute of Mental Health
