# Supplementary material for: Designing a Clinical Decision Support Tool That Leverages Machine Learning for Suicide Risk Prediction: Development Study in Partnership With Native American Care Providers
Source: JMIR Public Health Surveill. 2021 Sep 2;7(9):e24377. doi: 10.2196/24377 (PMC8446841; doi:10.2196/24377)
Supplement: Multimedia Appendix 1 [file publichealth_v7i9e24377_app1.docx]

**Understanding and responding to risk in Celebrating Life**

*Interviews will be loosely structured. Specific wording of questions and the order in which they are introduced will differ between participants. The following is a general guide for the questions that will be brought up in each interview.*

Thank you for agreeing to participate in an interview. The interview will take approximately 30 minutes. Your insights will help us determine how risk flags may be implemented in CL and tailored to assist case managers.

**Questions about practice and experience:**

- **How long have you worked on the CL team?**
- **What does your typical day look like?**
- **When working with someone in the CL registry, how do you know when someone is at risk of actually committing suicide? How do you know when someone is not at risk?**
- **How do you know when someone is at immediate risk? How do you know when someone is chronically at risk?**

**For the following questions, please think of what CL can do that is feasible given existing resources.**

- **What can be done for someone…**
  - **At immediate high risk?**
- **At immediate medium risk?**
- **At immediate low risk?**
- **What about someone who is more at chronic risk. What can be done for someone at…**
- **At chronic low risk**
- **At chronic** **medium risk**
- **At chronic high risk**
- **What factors do you consider when assessing for suicidality?**

**We have developed a way to flag people in the CL system who are at higher risk of suicide or suicide attempt based on their reported behaviors. This can flag people at risk for these outcomes 12 and 24 months from their first encounter with the CL system (first yellow form). However, we need your help in thinking through how to implement this so its useful.**

- **At what point in the CL system would you want people at risk to be flagged?**
- **How would flagging people at risk for suicide attempt or death 12 months from now help you?**
